# Effect of Load on the Thermal Expansion Behavior of T700 Carbon Fiber Bundles

**DOI:** 10.3390/polym10020152

**Published:** 2018-02-06

**Authors:** Guoliang Geng, Xiaofei Ma, Hongbin Geng, Yiyong Wu

**Affiliations:** 1 School of Materials Science and Engineering, Harbin Institute of Technology, Harbin 150001, China; genghongbin@hit.edu.cn (H.G.); wuyiyong@hit.edu.cn (Y.W.); 2 CAST-Xi’an Institute of Space Radio Technology, Xi’an 710100, China

**Keywords:** T700 carbon fiber bundles, thermal cycles, coefficient of thermal expansion, microstructural analysis

## Abstract

T700 carbon fiber bundles (CFBs) are the primary material used for manufacturing cable-net in a deployable antenna. In this paper, the relationships between the coefficient of thermal expansion (CTE) of T700 CFBs and the experimental load were investigated. The microstructure of T700 CFBs was analyzed with Raman spectra and XRD before and after the thermomechanical test. The measured results indicated that the T700 CFBs that were parallel to the axis had negative expansion characteristics when in a temperature range of −150–+150 °C. The thermal strain that occurred during the heating and the cooling thermal cycles had an unclosed curve that served as the loop. When the thermal cycles were the same, the position of the strain loop and the length of the sample exhibited regular change. The average of the CTEs decreased as the experimental load increased. The microstructural analysis suggested that the degree of structural order and the degree of orientation along the fiber axis improved with the experimental load increase. The change of microstructure parameters could be the primary cause of the negative CTE’s variation within the T700 CFBs. The experimental results provide some guidelines for improving the cable-net material selection.

## 1. Introduction

The deployable communications antenna is a key spacecraft subsystem component for various mission applications, including deep-space probes, reconnaissance, communications, and environmental monitoring. The antenna plays an essential role in modern aerospace applications. The mesh reflector deployable antenna is a typical deployable antenna that works in orbit. This antenna has a low mass, a simply configuration, and a low surface distortion [1,2,3,4]. The cable-net (including the front net, rear net and vertical cables) is the most important component in the deployable antenna, where it plays a significant role in the shaping and the use of the antenna. At present, the research of cable-net is mainly focused on the simulation calculation and analysis and optimization of the cable-net structures. Such as Zhang et al. [5] present a cable-net tension analysis method during the deployment of deployable antennas, the validity of this method has been verified by experimental results. Nie et al. [6] proposes the parameterized deployment analysis for space cable net structures considering geometric nonlinearity and topological diversity, this provides a general and effective way to analyze the deployment of space cable net structures with various topologies and parameters. However, there is a little research on the performance of cable-net under actual working conditions. On the other hand, the working environment of antenna is very complicated, which puts forward stricter requirements for material that manufacture the cable-net. Carbon fiber is known for its superior mechanical properties, its light weight, and its high temperature tolerance [7,8,9,10,11]. These excellent properties make carbon fiber the most suitable material for manufacturing cable-net, which is primarily composed of T700 carbon fiber bundles (CFBs) in practical engineering applications.

When a satellite antenna moves around the earth, for example, going repeatedly into and out of the shadow of the earth, its surface temperature varies in a wide range according to the orbit altitude. For a satellite moving in low orbit from the earth at periodic return of 90 min, its surface temperature varies between −101 and 93 °C [12]. Meanwhile, due to the wide range of environmental temperature variation, the various components working on the satellite antenna will also have a great impact. For example, the cable-net will be affected by the thermal strains or the load-induced strain that occur during the use of deployable antennas. These impacts degrade the radiation pattern with lower gain, higher side-lobe level, and beam shift [13,14]. It is crucial to determine the thermal expansion behavior in the cable-nets and guarantee the shape precision of the reflector, in order to ensure good work performance.

Thermal expansion behavior is closely related to the coefficient of thermal expansion (CTE) within practical engineering applications. The linear thermal expansion coefficient, α, is defined as the ratio between the thermal strain change to the temperature change: Δε/Δ*T*. One of the more unusual properties of carbon fibers is its strong anisotropic ability, with respect to the coefficient of thermal expansion (CTE). Carbon fibers have a small negative CTE in the longitudinal direction (parallel to the axial direction of fiber) and a positive CTE in the transverse or radial direction [15]. This means that the carbon fibers have a negative α as the temperature increases. This increase is caused by the increased rotation surrounding the Carbon-Carbon bonds in the chain direction [16,17]. Previous literature has suggested that this mechanism is a general feature of crystalline polymers [18].

Current studies concerning the thermal expansion behavior or the CTE of carbon fiber have focused on the single filaments of the carbon fibers. Sauder et al. [19] evaluated the tensile behavior of carbon fibers at temperatures up to 2400 °C. Pradere et al. [20] also measured the thermal properties of carbon fibers at high temperatures (up to 2500 K). There has been little research on carbon fiber bundles (CFBs), especially in different experimental conditions (such as various experimental loads or thermal cycles). Previous literature has addressed the influence of applying a simplex load on the thermal expansion behavior of carbon fibers, or has addressed the heating or the cooling process, while ignoring the thermal cycle conditions.

In this paper, the thermal expansion behavior and CTE of T700 CFBs were investigated by using the tensile loads as the variables. This will provide insight for the effective prediction of thermal expansion behaviors of the T700 CFBs, under thermal cycling conditions. The research will also provide a guideline and a method for improving the thermal stability of the cable-net, and optimizing the selection of materials and design of cable-net. The regressive model equation was proposed between the CTE of the T700 CFBs and the experimental loads. This model equation has great significance for practical engineering applications. 

## 2. Experimental Procedure

### 2.1. Materials and Specimen Preparation

**Figure 1:**
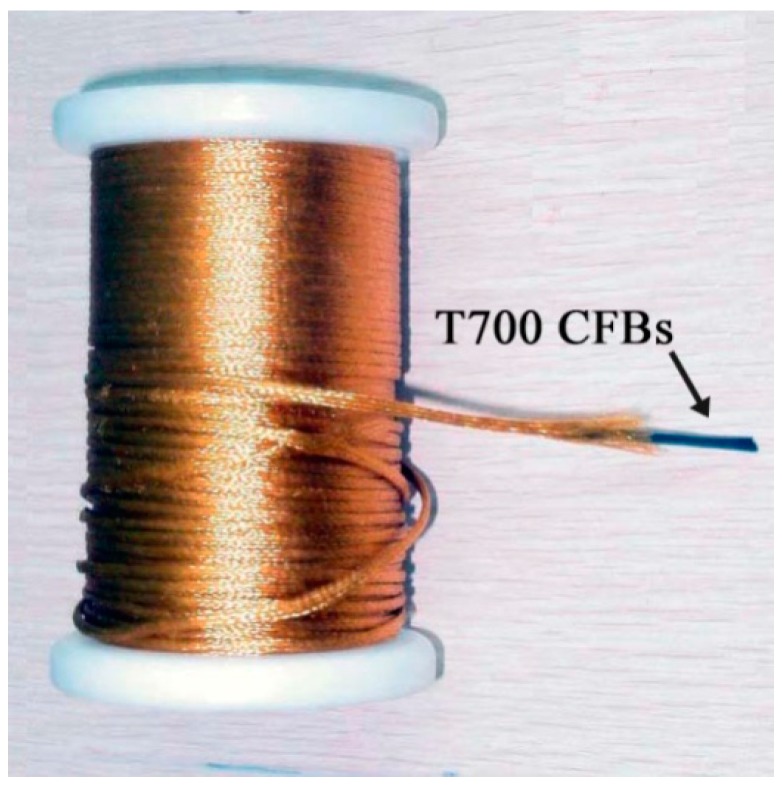
The physical characteristics of T700 CFBs.

The T700 CFBs (Figure 1) were composed of PAN-based T700SC parallel fiber bundles, including 12 K filaments fabricated by Toray Industries, Inc., Tokyo, Japan. The nominal modulus and tensile strength of T700 are 230 GPa and 4900 MPa respectively, and the nominal diameter for the sample of bundles was 1.4 mm, with an original gauge length of 500 mm at 1 N tensile load. All samples were dried at 50 °C for 3 h.

The specimen should be loaded with the corresponding load during the thermal expansion test, and must keep in a vertical and tight state. The pre-stretching treatment is required before the thermal expansion test for all the specimens to ensure that the initial state of the specimen is the same under different load conditions, because of some influences of internal factors within CFBs (such as knots and twists inside the CFBs, or the strain relaxation characteristic of the CFBs). The pre-stretching treatment was performed at the Thermo-Mechanical Analyzer (TMA) (This instrument is self-made by Harbin Institute of Technology, Harbin, China), and the samples were pre-stretched at room temperature by periodically cycling the tension from 0 to 80 N 30 times for 500 s. When the length of the sample tended to be stable, the thermal expansion was measured. The sample in the pre-stretching treatment and actual test was the same one.

**Figure 2:**
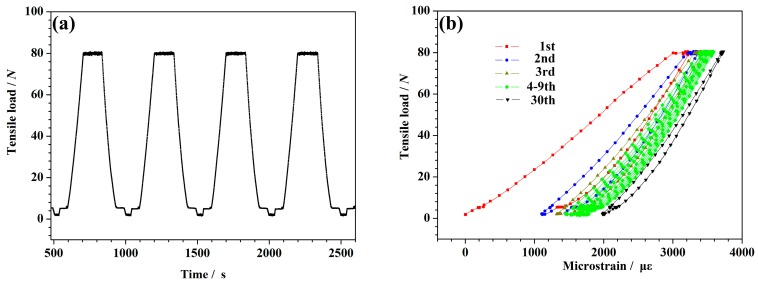
(**a**) The diagram of typical pre-stretching curves between tensile load and time. (**b**) The diagram of typical pre-stretching curves between tensile load and microstrain.

The time history curve of the tensile load during pre-stretching is shown in Figure 2a. Figure 2b illustrates the relationship between the tensile load and the microstrain during pre-stretching. Figure 2b shows that the microstrain curve exhibited a hysteresis loop that was a typical dynamic viscoelasticity, resulting in strain relaxation. In the initial stage of pre-stretching, the microstrain of the CFBs showed a significant change, which was an obvious strain relaxation characteristic. The microstrains of the CFBs decreased and tended toward stability as the pre-stretching durations increased. The initial microstrain values, under the various load conditions, are provided in Table 1.

### 2.2. Measurement of Coefficients of Thermal Expansion

**Figure 3:**
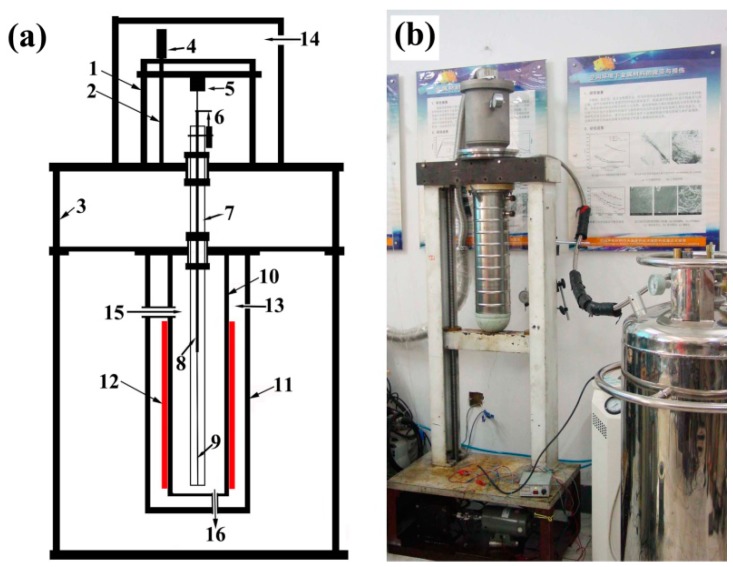
(**a**) Schematic diagram of the Thermo-mechanical analyzer (1. lead rail; 2. ball screw; 3. the frame; 4. loading motor; 5. load sensor; 6. strain sensor; 7. the benchmark frame of quartz tube; 8. quartz rod; 9. the sample; 10. thermal conductivity layer; 11. the lagging; 12. infrared lamp; 13. liquid nitrogen; 14. the interface of vacuum and argon gas; 15. the inlet of argon gas; 16. the outlet of argon gas) and (**b**) The picture of the test facility for the measurement of coefficients of thermal expansion.

The measurements were performed with a TMA (The schematic diagram and the picture of the test facility for the measurement of coefficients of thermal expansion. are shown in Figure 3) in a pure argon atmosphere. This was chosen based on the inductive dilatometer principle [21,22,23]; see investigation reference GJB332A-2004 (test method for linear expansion coefficient of solid materials). The argon gas flux was accurately controlled with a mass flow controller. This measurement technique used a linear variable differential transformer (LVDT), (Beijing Wavespectrum Science and Technology Co., Ltd., Beijing, China) where a quartz crystal was attached to a quartz tube that formed a section of the LVDT core. The thermal cycle was the sequence from ambient temperature, heated by an infrared lamp up to 150 °C, cooled with liquid nitrogen down to −150 °C, and then allowed to return to room temperature.

The controlled rate of heating (or cooling) of the specimen was about 3–5 °C per minute. The specimen was then placed in a quartz tube frame. The top end of the specimen was fixed with the frame and the opposite end was fixed with a quartz rod that formed a section of the LVDT that was attached to measure the expansion. A software program (This software is developed by Harbin Institute of Technology, Harbin, China) controlled the test parameters and the temperature was continually measured along with the strain data during the heating and the cooling cycles. The CTE was obtained from the linear fit of the data over the entire test-temperature range.

The thermomechanical load induced deformation of the CFBs, which consisted of a reversible and an irreversible component. The reversible component was directly related to the coefficient of the carbon fiber thermal expansion. The irreversible deformation was associated with the changes in the microstructure, which resulted in the creep deformation. The negative CTE was a mixed creep deformation interwoven together with the materials, during the thermomechanical test. The cooling cycle tended to increase the measured deformation. The heating cycle tended to decrease the measured deformation. The mean α values were calculated by averaging the values of the slopes that corresponded to the heating and the cooling cycles. This procedure could reduce and compensate for the creep deformation induced error. The expansion coefficient of the cooling stage and the heating stage were measured. The mean values for both were used as the thermal expansion coefficient of the sample. 

### 2.3. Characterization

The microstructure of T700 CFBs was measured with Raman spectroscopy, X-ray diffraction (XRD), and transmission electron microscopy (TEM).

Raman spectroscopy was performed with a Horiba Jobin-Yvon HR800 confocal laser micro-Raman spectrometer (HORIBA Jobin Yvon, Paris, France) that had a 458 nm argon ion laser. This measured the quality and the degree of structural order in the carbon fiber from the surface and the cross-section. The Raman spectrometer was placed in the continuous scanning mode, with a 20 mW laser beam power, and ×50 magnification objective lens. The focused laser beam was 1 μm in diameter, the Raman shifts were calibrated with a 520.7 cm^−1^ line of a silicon wafer prior to the experiment, and a 30 s accumulation time was used throughout the measurements. The Raman data was curve-fitted using a PeakFit V4.12 to obtain the peak positions. The full-width at half maximum (FWHM) of D peak and G peak was fitted with the Gauss add Lorentz fitting.

The crystalline structures of the T700 carbon fiber bundles were analyzed with an X-ray diffraction (XRD) instrument (PANalytical X’Pert Pro, Almelo, The Netherlands) using Cu-*K*α radiation (λ = 0.1541 nm) before and after the thermal cycles. The working voltage and the current of the target were 40 kV and 40 mA. Each sample was scanned over a 2θ range from 10° to 90°, in 0.05° increments. Measurements were performed with an equatorial scan and an azimuthal scan at the fixed Bragg position. The diffraction curves were fitted with the MDI Jade 6.0 (MDI, Livermore, CA, USA). The structural parameters were obtained with the Bragg equation and the Scherrer formula.

The TEM sample was cut into a relatively thin section using a focused ion beam (HELOIS NanoLab600i, Hillsboro, OR, USA). The TEM patterns were obtained with a Talos F200x microscopy that had a 200 kV CCD detector.

## 3. Results and Discussion

### 3.1. Effect of Experimental Loads on Thermal Expansion Behavior of Cfbs

**Figure 4:**
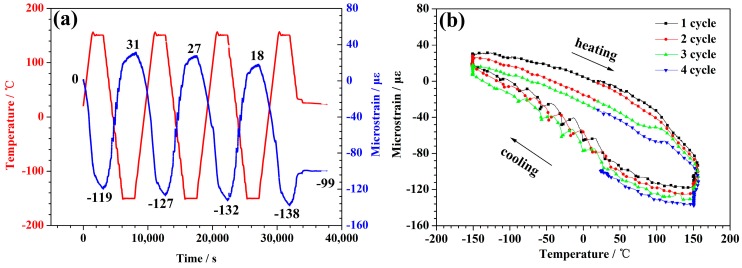
The thermal expansion behavior for the applied 0.05 N sample that underwent thermal cycling. (**a**) The plots of the temperature and the micro-strain as a function of time. (**b**) The change in the axial strains versus the temperature.

Figure 4a depicts the temperature curves and the deformation rate for the specimen with experimental load 0.05 N. The sample contracted as the temperature increased, and expanded as the temperature decreased. This indicated that the CTE of the CFBs was negative throughout the measured temperatures. There was a slight decrease in the length as the thermal cycles increased. The length of the specimen shrank 99 microstrains after 3.5 cycles. The average of shrinkage was about 28 microstrains per cycle.

As is shown in Figure 4a, the red curve is the temperature-time curve and the blue curve is the microstrain-time curve. In order to investigate the relationship between temperature and microstrain directly, we converted the data from Figure 4a to obtain the relationship diagram (see Figure 4b) between the deformation and the temperature under the experimental load 0.05 N. Figure 4b shows that the microstrain curves of the samples were different during the heating and the cooling processes. With the increase of thermal cycles, the microstrain curves showed the pattern of multiple hysteresis loops, and the hysteresis loop constantly moved down. This corresponded to the shortening of the sample mentioned in the previous paragraph.

**Figure 5:**
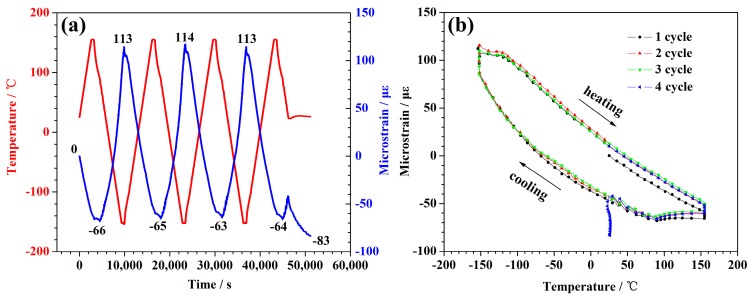
The thermal expansion behavior for the applied 2.5 N sample that underwent thermal cycling. (**a**) Plots of the temperature and the microstrain as a function of time. (**b**) Changes in the axial strains versus the temperature.

Figure 5a describes the relational curves of the temperature-time and the microstrain-time under 2.5 N. Figure 5a shows that the sample contracted as the temperature increased, and expanded as the temperature decreased. This pattern indicated that the CTE of the CFBs was negative throughout the −150–+150 °C temperature range under 2.5 N. The length of the sample was nearly unchanged before and after the test. The variation of the sample was 83 microstrains after 3.5 thermal cycles.

Similar to Figure 4b, the strain curve was the shape of the multiple hysteresis loops. Unlike Figure 4b, the position of the hysteresis loop did not move with the 2.5 N experimental load. The hysteresis loop of with 3.5 cycles nearly coincides with the initial hysteresis loop. It has been shown that the length of the sample before and after the thermal cycle was nearly unchanged under the 2.5 N experimental load.

**Figure 6:**
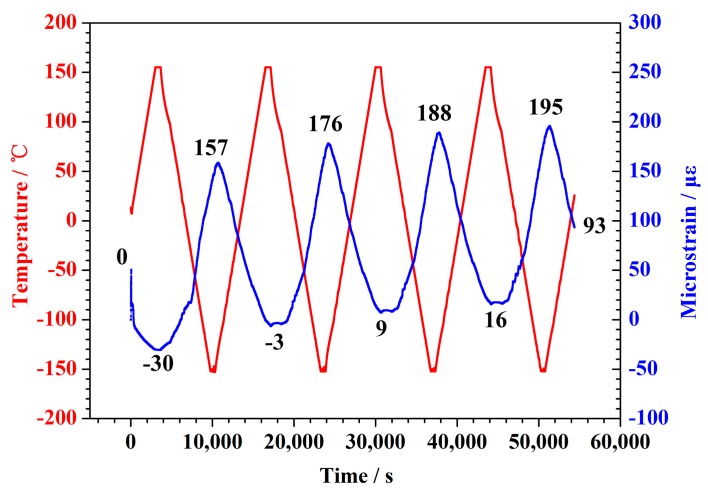
Plots of temperature and microstrain as a function of time for the 5 N load sample.

The temperature-time curves and microstrain-time curves at the 5 N experimental load are shown in Figure 6. The specimen shortened with the temperature increased, and expanded as the temperature decreased. This indicated that the CTE of the CFBs was negative throughout the −150–+150 °C temperature ranges. The length of the specimen increased slightly under the 5 N load after 4 thermal cycles, this point different from 0.05 and 2.5 N specimens. The expansion value in length of specimen was 93 microstrains in the final state. The average was about 23 microstrains per cycle.

**Figure 7:**
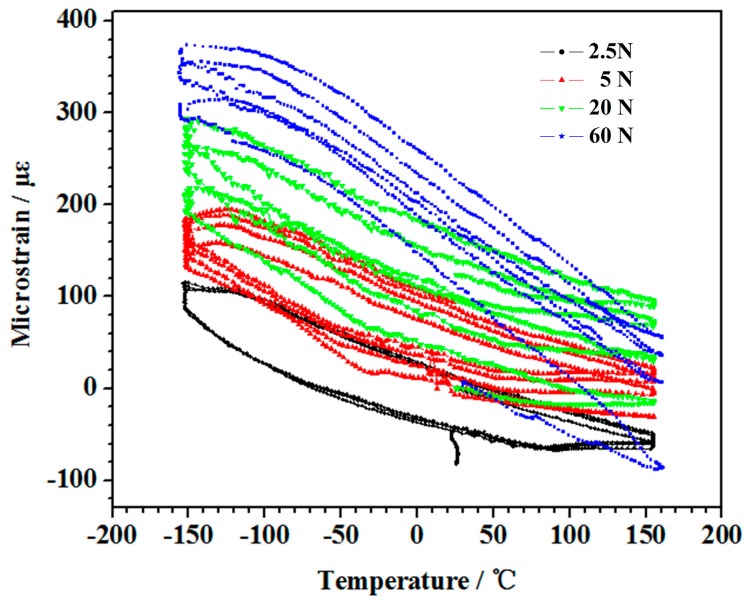
The change in axial strains for the applied series load samples that underwent thermal cycling.

Figure 7 shows the relationship between the microstrain and the temperature under different experimental loads. The effect of the load on the deformation of the thermal expansion was explored by plotting, the microstrain curves of the 2.5, the 5, the 20, and the 60 N loads, see Figure 7.

Figure 7 shows that the microstrain curves all obtained the multiple hysteresis loops pattern. When the experimental load was greater or equal to 5 N, the microstrain curves continuously moved up. The amount of deformation increased with the increased experimental load. This result demonstrated that the average of the CTE decreased as the experimental load increased. This trend became more obvious as the load changes were larger. The CTEs of the samples that underwent 3 thermal cycles are depicted in Table 1.

The cable-net is an important component of deployable antennas, where it plays a significant role in the shaping and the use of the antenna. However, the thermal expansion behavior of T700 CFBs is a chief cause for deformation of cable-net. It is necessary to provide a prediction technique for the deformation of cable-net and reference data for the design and the adjustment in ground test of cable-net. Establishing the degenerate model formula between the load and the CTE of T700 CFBs was the most effective method between the temperature ranges between −150–+150 °C.

**Figure 8:**
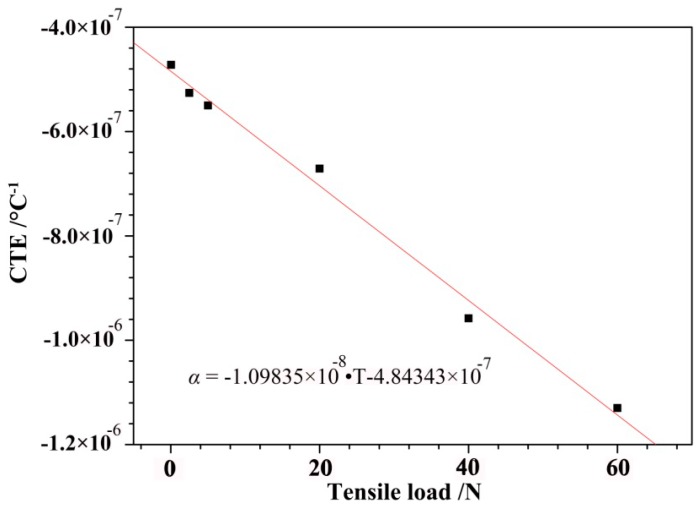
The empirical equation of the CTEs that corresponded to the applied series loads.

Figure 8 depicts the relationship between the CTE and the applied series load. Figure 8 shows that the CTE decreased linearly as the experimental load increased. The data presented in Table 1 shows that the estimated regression line was represented by the following equation:

α = (−1.09835 × 10^−8^) × *T* − (4.84343 × 10^−7^) (1)

where α was the CTE of the sample (°C^−1^), and *T* was the tensile load (N). The computational error of calculated results from the degenerate model formula was less than 3.5 × 10^−8^.

**Figure 9:**
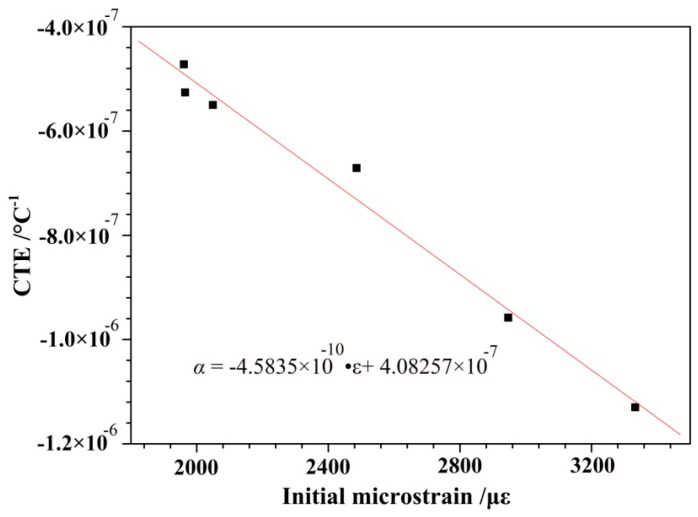
The empirical equation of the CTEs that corresponded to the initial microstrain.

A degenerate model formula was set up between the initial microstrains and the CTE of T700 CFBs. The relationship between the CTE and the initial microstrain is shown in Figure 9. The equation was rewritten as a function of the initial microstrain, as follows:

α = (−4.5835 × 10^−10^) × ε + (4.0825 × 10^−7^) (2)

where α was the CTE of the sample (°C^−1^), and ε was initial strain expressed in μ.

As shown in Figure 9, the CTE exhibited a tendency of linear decline as the initial microstrain increased. A comparison of the data between the 0.05 N load and the 60 N load showed that the corresponding CTE increased about 2.4 times, while the initial microstrain increased about 1.7 times. This shows that even a slight change in the initial microstrain would have a large effect on the CTE of T700 CFBs. This should be taken into account during testing.

In practical engineering applications the repeated complex tests and the estimated test results should be avoided. It is most effective and direct to establish the degradation model formula based on the measured data. The degradation model formula for the CTE of T700 CFBs in the temperature range between −150–+150 °C provided an effective prediction method for calculating thermal expansion deformation of cable-net. This method has great significance in engineering applications.

### 3.2. Microstructural Analysis of Cfbs before and after the Thermomechanical Test

Raman spectroscopy is a nondestructive technique that is widely used to characterize the structural and the electronic properties of carbon materials, including carbon nanotubes, diamond, graphite, and carbon fibers [24,25,26]. The carbon materials in the first order spectrum (1000–2000 cm^−1^) showed two primary characteristic bands (the G band at 1580 cm^−1^ and the D band at 1360 cm^−1^). It is widely accepted that within the Raman spectra of the carbon materials G band’s position, the G band’s FWHM and the ratio of the integrated intensities of D band to G band (*R* = *I*_D_/*I*_G_) could be used to evaluate the degree of structural order and the crystalline structure of carbon materials [27,28,29,30]. The positional change of the G band had a complex relationship with the experimental temperature and load. The degree of the structural order of carbon materials improved when the G band’s FWHM became narrower and the value of R became smaller [31,32,33]. In this paper, *υ* and *W* represented the peak position and the full width at half maximum.

**Figure 10:**
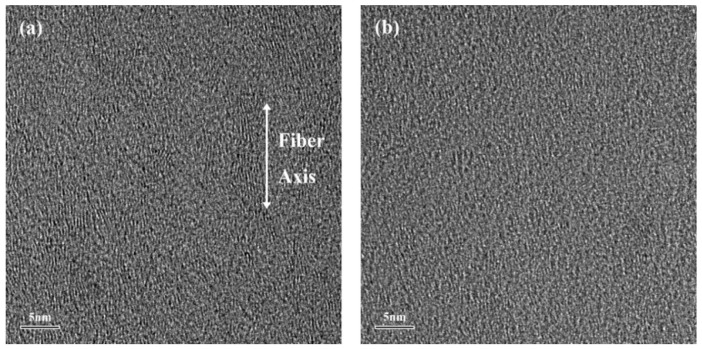
TEM images in the longitudinal section of the initial sample (**a**) near the surface (**b**) and near the core.

Figure 10 shows the TEM pattern near the surface and near the core of the T700 carbon fibers. The structure was different between the surface region and the core region of the T700 carbon fibers. The surface of the fibers displayed a well-order graphite structure, but the core of the fibers exhibited random micro-textures and a graphite structure. The microstructure near the surface exhibited little change before and after the thermomechanical test (60 N experimental load over 240 cycles). The Raman data near the core was used as the correlation data. The expression *I*_D_/(*I*_D_ + *I*_G_) was used to evaluate the relative content of the microcrystalline defects in the carbon materials [34]. 

X-ray diffraction (XRD) is the most common technique used for quantitative structure analysis at a molecular length scale. The 002 peak obtained from the equatorial scan was used to estimate the average interlayer spacing values (*d*_002_) and the apparent crystallite thickness (*L*_c_). The *d*_002_ value was calculated with the Bragg formula and the crystallite size (*L*_c_) was calculated with the Scherrer equation.

The degrees of orientation of the graphite layer planes parallel to the fiber axis were obtained by the azimuthal scan at the fixed Bragg position (002 reflection). The π and *Z* represented the orientation degree of the carbon layer and the orientation angle. Theoretically, the structural parameter of the carbon materials would be better with a smaller *d*_002_ and a larger *L*_c_ [35,36,37]. 

**Figure 11:**
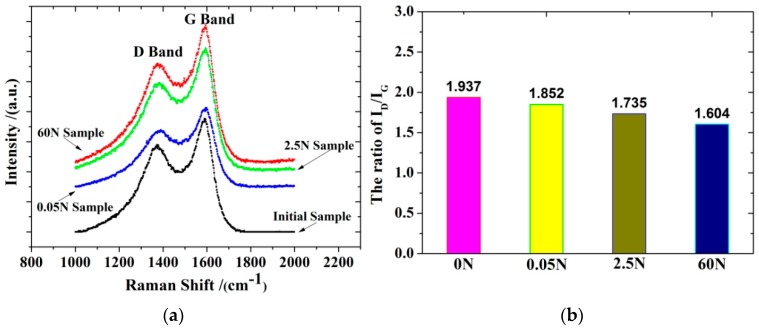
(**a**) The Raman spectra of the sample at the various experimental loads and (**b**) the relation diagram between the *I*_D_/*I_G_* ratio and various experimental loads.

Figure 11a and Table 2 shows that the G band’s peak shifted towards higher frequencies during the first stage. As the experimental load increased, the G band’s peak gradually shifted towards lower frequencies. This variation was consistent with the microstrain change of the sample in the thermomechanical test. 

The FWHM width of the G band and the relative content of the microcrystalline defects gradually reduced as the experimental load increased; see Table 2 and Figure 11b. The value of R, which estimated the degree of structural order for carbon materials, also decreased as the load amount increased. This indicated that the crystalline structure improved. The degree of structural order for the CFBs improved as the experimental load increased from 0 to 60 N.

**Figure 12:**
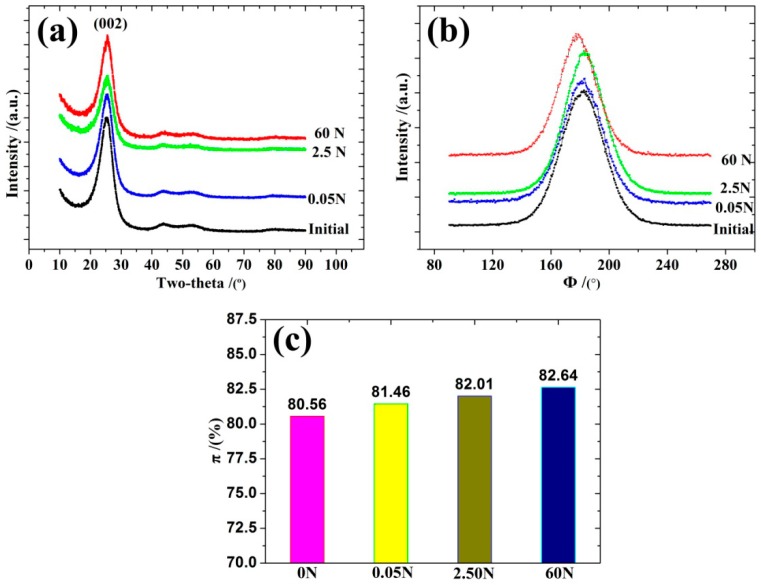
(**a**) XRD patterns for various experimental load samples via an equatorial scan. (**b**) The XRD patterns for the various experimental load samples via an azimuthal scan. (**c**) The relation diagram between the degree of orientation and the various experimental loads.

The XRD patterns of various experimental load samples are shown in Figure 12. The microstructure parameters are listed in Table 3. The structural evolution was demonstrated by the change of the shape and the position. The FWHM for the diffraction peaks are displayed. The equatorial scan patterns (Figure 12a and Table 3) showed that the *d*_002_ exhibited a monotonic decrease, while the *L*_c_ increased when the experimental load increased from 0 to 60 N.

The microstructure results obtained with the XRD were consistent with the results obtained by the Raman spectra. The degrees of structural order, the degree of orientation, and the microcrystalline structure of T700 CFBs improved as the experimental load increased. This was in agreement with previous literature, which stated that the degrees of structural order and the degree of orientation of carbon fibers have a close relationship with the CTE of the carbon fibers [11,38]. As the load increased further, additional crystallites became parallel to the fiber axis and the orientation of crystallites became more consistent. This could be the cause of the regular changes that occurred in the CTE of T700 CFBs as the experimental load increased.

## 4. Conclusions

In this work, the thermal expansion behavior of T700 CFBs was investigated by the Thermo-Mechanical Analyzer. Especially, we focused on the effect of different experimental loads on the CTE of T700 CFBs and the deformation rule of thermal expansion when thermal cycles were the same. The following conclusions were drawn:

(1) The T700 carbon fiber bundles that were parallel to the axis exhibited negative expansion characteristics within a temperature range between −150–+150 °C.
(2) The strain curves of the specimen during the heating and the cooling cycles emerged as hysteresis loops during the thermal cycles. As the experimental load increased, the position of hysteresis loops exhibits a tendency to move down firstly and then move up gradually. The length of the sample also shows the state of shortening firstly and then elongating gradually.
(3) The averages of the CTEs of T700 CFBs decreased gradually as the load increased, and the empirical approach for the prediction of the changes in the CTE can be expressed as an exponential relationship between the extent of variation and the experimental load.
(4) The Raman spectra and the XRD analysis showed that the microstructural parameters of the T700 CFBs changed as the experimental load increased. This could be the primary cause for the negative CTE’s variation in the T700 CFBs.

## Figures and Tables

**Table 1:** The axial CTEs of the samples applied series load during the three thermal cycles and the series test load that corresponded to the initial microstrain.

Test Loads (N)	Test Status	Tested Results (°C^−1^)			Average (°C^−1^)	Initial Microstrain (με)
		First Time	Second Time	Third Time		
0.05	Cooling	−5.30 × 10^−7^	−5.42 × 10^−7^	−5.32 × 10^−7^	−4.72 × 10^−7^	1961
	Heating	−3.65 × 10^−7^	−4.36 × 10^−7^	−4.29 × 10^−7^		
	Mean	−4.47 × 10^−7^	−4.89 × 10^−7^	−4.81 × 10^−7^		
2.5	Cooling	−4.79 × 10^−7^	−4.67 × 10^−7^	−4.60 × 10^−7^	−5.26 × 10^−7^	1965
	Heating	−5.77 × 10^−7^	−5.86 × 10^−7^	−5.84 × 10^−7^		
	Mean	−5.28 × 10^−7^	−5.27 × 10^−7^	−5.22 × 10^−7^		
5	Cooling	−5.06 × 10^−7^	−4.97 × 10^−7^	−4.89 × 10^−7^	−5.50 × 10^−7^	2049
	Heating	−5.85 × 10^−7^	−6.09 × 10^−7^	−6.13 × 10^−7^		
	Mean	−5.45 × 10^−7^	−5.53 × 10^−7^	−5.51 × 10^−7^		
20	Cooling	−6.97 × 10^−7^	−6.77 × 10^−7^	−6.35 × 10^−7^	−6.71 × 10^−7^	2486
	Heating	−6.49 × 10^−7^	−6.76 × 10^−7^	−6.95 × 10^−7^		
	Mean	−6.73 × 10^−7^	−6.76 × 10^−7^	−6.65 × 10^−7^		
40	Cooling	−1.14 × 10^−6^	−1.11 × 10^−6^	−1.08 × 10^−6^	−9.58 × 10^−7^	2947
	Heating	−7.97 × 10^−7^	−8.04 × 10^−7^	−8.20 × 10^−7^		
	Mean	−9.67 × 10^−7^	−9.56 × 10^−7^	−9.51 × 10^−7^		
60	Cooling	−1.23 × 10^−6^	−1.13 × 10^−6^	−1.09 × 10^−6^	−1.13 × 10^−6^	3333
	Heating	−1.08 × 10^−6^	−1.13 × 10^−6^	−1.13 × 10^−6^		
	Mean	−1.15 × 10^−6^	−1.13 × 10^−6^	−1.11 × 10^−6^		

**Table 2:** The structural parameters of the T700 CFBs over various experimental loads measured with Raman spectra.

Sample	*ν*_D_/cm^−1^	*ν*_G_/cm^−1^	*W*_D_/cm^−1^	*W*_G_/cm^−1^	*I*_D_/(*I*_D_ + *I*_G_)
Initial	1372	1590	266	109	0.6595
0.05 N	1390	1595	260	107	0.6494
2.5 N	1383	1590	258	106	0.6344
60 N	1374	1589	217	103	0.6160

**Table 3:** The structural parameters measured with the XRD for the sample subjected to various loads.

Sample	2θ/°	*d*_002_/nm	*L*_c_/nm	*X*_c_/%	*Z*/°
Initial	25.527	0.3488	1.6901	66.79	17.4935
0.05 N	25.577	0.3480	1.6962	68.93	16.6855
2.5 N	25.624	0.3474	1.7188	70.45	16.1975
60 N	25.749	0.3458	1.7290	73.27	15.6220
